# Wellbeing and resilience: mechanisms of transmission of health and risk in parents with complex mental health problems and their offspring—The WARM Study

**DOI:** 10.1186/s12888-015-0692-6

**Published:** 2015-12-09

**Authors:** Susanne Harder, Kirstine Davidsen, Angus MacBeth, Theis Lange, Helen Minnis, Marianne Skovsager Andersen, Erik Simonsen, Jenna-Marie Lundy, Maja Nyström-Hansen, Christopher Høier Trier, Katrine Røhder, Andrew Gumley

**Affiliations:** Department of Psychology, University of Copenhagen, Copenhagen, Denmark; Department of Child and Adolescent Mental Health Odense, Research Unit, Mental Health Services in the Region of Southern Denmark, Middelfart, Denmark; University of Southern Denmark, Odense, Denmark; School of Health in Social Science, University of Edinburgh, Edinburgh, Scotland UK; Department of Public Health, Section of Biostatistics, University of Copenhagen, Copenhagen, Denmark; Institute of Health and Wellbeing, University of Glasgow, Glasgow, Scotland UK; Psychiatric Research Unit, Psychiatry, Region Zealand, Roskilde, Denmark; Institute of Clinical Medicine, Faculty of Health and Medical Sciences, University of Copenhagen, Copenhagen, Denmark

**Keywords:** High-risk infants, Risk development, Schizophrenia, Bipolar disorder, Depression, Cohort study, Attachment, Stress-sensitivity, Caregiving

## Abstract

**Abstract:**

The WARM study is a longitudinal cohort study following infants of mothers with schizophrenia, bipolar disorder, depression and control from pregnancy to infant 1 year of age.

**Background:**

Children of parents diagnosed with complex mental health problems including schizophrenia, bipolar disorder and depression, are at increased risk of developing mental health problems compared to the general population. Little is known regarding the early developmental trajectories of infants who are at ultra-high risk and in particular the balance of risk and protective factors expressed in the quality of early caregiver-interaction.

**Methods/Design:**

We are establishing a cohort of pregnant women with a lifetime diagnosis of schizophrenia, bipolar disorder, major depressive disorder and a non-psychiatric control group. Factors in the parents, the infant and the social environment will be evaluated at 1, 4, 16 and 52 weeks in terms of evolution of very early indicators of developmental risk and resilience focusing on three possible environmental transmission mechanisms: stress, maternal caregiver representation, and caregiver-infant interaction.

**Discussion:**

The study will provide data on very early risk developmental status and associated psychosocial risk factors, which will be important for developing targeted preventive interventions for infants of parents with severe mental disorder.

**Trial registration:**

NCT02306551, date of registration November 12, 2014.

## Background

Children of parents diagnosed with complex mental health problems including schizophrenia, bipolar disorder and depression, are at increased risk of developing mental health problems compared to the general population. Having one parent with schizophrenia results in a 7 % lifetime risk of schizophrenia [1] and 55 % risk of developing any psychiatric condition [2]. Having one parent with bipolar disorder results in a 6 % risk of bipolar disorder and a 60 % risk of any psychiatric condition, whereas for offspring of depression the risk for depression is 26 % and for any psychiatric condition 57 % [2]. Importantly despite this increased risk a large minority of these infants have a resilient development. Development of mental health problems starts during childhood and elevated levels of childhood mental health difficulties including externalizing disorders and ADHD have been reported in high-risk offspring [3, 4]. Within a developmental psychopathology framework, these child and adolescent disorders may themselves represent staging posts towards further mental health difficulties in early adulthood. In addition developmental vulnerability factors for adverse outcomes have been observed in multiple domains from preschool age, including socioemotional, cognitive, neuromotor, language, and psychopathological factors [5]. Children of parents with non-affective psychosis and depression display more cognitive and emotional problems preschool, and difficulties of social adjustment at school age [6]. The lifetime personal, familial, societal and financial costs associated with elevated risk have been established as a significant global mental health burden [7]. Therefore prevention of severe mental illness (SMI) is an important societal priority.

Multifactorial models of risk have been proposed for explaining transmission of risk from parent to offspring of parents with non-affective psychosis [5] bipolar disorder [8], depression [9], and with broader diagnoses of SMI [10]. Similar multifactorial resilience models for young infants with mentally ill parents have also been proposed [11]. These theoretical models hypothesize that mental disorders are heterogeneous conditions that arise from the additive and interaction effects of multiple genetic and environmental risk and resilience factors at different phases of development. In line with this understanding several lines of research [12, 13] have proposed that the study of mental illness should not be limited to one diagnostic category as several diagnoses share genetic and environmental factors. Furthermore risk exposure and risk development starts during pregnancy [4, 5] and early signs of risk development towards mental illness may be less diagnosis specific than closer to the onset of the illness. Early risk development sets the infant on a developmental risk trajectory that might be more difficult to change later down the developmental path. Therefore, a primary prevention of psychopathology strategy should preferably start from pregnancy and could in the early years be framed in terms of promoting resilience and opportunities for improved development [11]. More knowledge on very early environmental transmission mechanisms and developmental outcome in high risk infants is important in order to guide such early preventive intervention programs. During the early years the infant is totally dependent on the caregivers, and infant risk development cannot be understood independent of the child-caregiver-interaction system. Environmental risk and resilience factors associated with infant-caregiver interaction is thus of pivotal importance in assessing very early risk development. This is supported by findings that growing up in an institution, degree of illness severity of the ill parent, emotional climate in the family and experiences of childhood trauma are important risk factors for severe psychopathology [6, 14, 15]. Based on these considerations the present study focuses on exploring mechanisms of transgenerational transmission and early developmental risk in infants of parents with severe mental illness.

### Objectives

The aims of the WARM study are:

1. To identify very early risk markers for non-optimal development in infants of mothers with severe mental illness.
2. To explore transmission mechanisms of risk from parent to infant focusing especially on three possible mechanisms: stress, maternal caregiving-representation and mother-infant interaction.

## Method

### Design

**Fig. 1:**
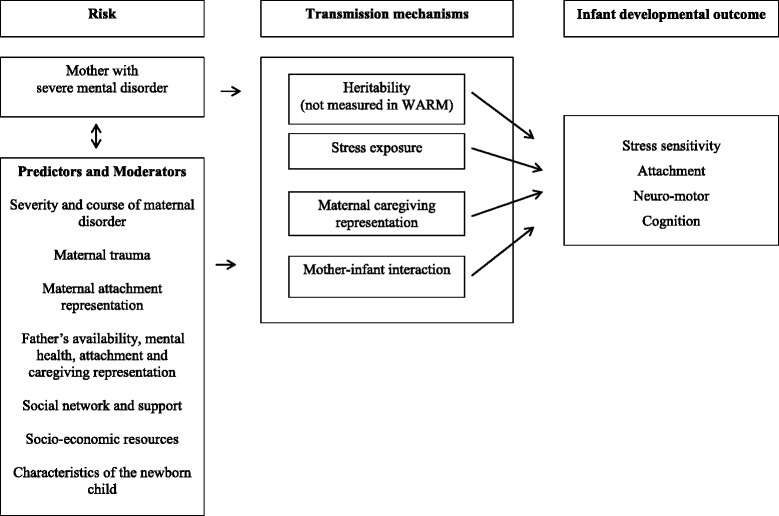
Transmission model

The WARM study is a Danish–Scottish prospective longitudinal cohort study following women with severe mental disorder (schizophrenia, bipolar disorder and severe depression), their partner and infant from pregnancy to infant 1 year of age. There are five assessment time points: during pregnancy and at 1–7 days, 4, 16 and 52 weeks of infant age. Fig. 1 illustrates the conceptual model underpinning the design of the study. The maternal mental illness constitutes the high risk status of the infant. Potentially predictors and moderators of transmission of risk are a) severity and course of maternal disorder, b) maternal trauma, c) maternal attachment representation, d) father/partner characteristics, e) support from social network, f) socio-economic resources of the family and g) characteristics of the newborn child [5, 9]. Heritability as a transmission mechanism is well established in severe mental disorder. The present study explores three possible environmental transmission mechanisms: stress, caregiving representation and mother-infant interaction. These are processes, which are suitable as targets for early preventive interventions. The infant outcome domains included in the study are stress-sensitivity, attachment, neuro-motor and cognitive development.

### Participants

The study includes pregnant women with first or subsequent pregnancies at a minimum age of 16 (in Scotland) and 18 (in Denmark) and older who are willing and able to provide informed and written consent to participate, provide informed and written consent for their unborn infant to participate and who meet the following inclusion criteria: a) lifetime DSM-5 Delusional Disorder, Schizophreniform Disorder, Schizophrenia or Schizoaffective Disorder, Psychosis NOS, Brief Psychotic Disorder or b) lifetime DSM-5 Bipolar I and II Disorder or c) DSM-5 Major Depressive Disorder (current moderate or severe single episode or lifetime recurrent moderate or severe) or d) a non-psychiatric control group defined as mothers without any history of treatment or admission for a psychiatric disorder or drug or alcohol addiction. Mothers describing current psychiatric symptoms not previously identified or treated and who are likely to require treatment will not be included. Exclusion criteria are: a) Unable to speak English or Danish; these are excluded because of the requirement to complete assessments, b) Miscarriage, c) Maternal diagnosis of Autistic Spectrum Disorder, d) Alcohol or drug dependency being the primary diagnosis. In the event that the women lose capacity to consent during participation in the study, maternal and infant involvement will be suspended until capacity is regained. The study is aiming at a sample size of 200 with 50 in each group, distributed proportionally between Denmark and Scotland.

Partners will be approached for their informed consent and will be eligible if they are: a) the biological parent of the infant and have a caregiving role in relation to the infant or, b) partners who have a caregiving role in relation to the infant but are not the biological parent of the infant or; c) another person nominated by the mother who is in a close caregiving role (e.g. grandmother).

Infants will be included in the study from birth, if the parents (mother in Scotland) having parental responsibility for the child have provided informed and written consent for their child to participate in the study. There are no a priori exclusion criteria for the infant.

### Assessments

The mapping of assessment domain to measurement is detailed in Table 1 and the schedule of measurements is illustrated in Table 2. All measures are validated.

**Table 1:** Mapping of domains to measurements

Risk	Measure	Person
Diagnosis	SCID	Mother
Personality disorder screen	SAPAS	Parents
Predictors & moderators		
Symptom severity	MADRS	Mother
	BRMRS	Mother
	PANNS	Mother
	BSI-53	Father
Trauma	ACES	Mother
Attachment	AAP	Parents
	AAI	Mother
	Psychosis Attachment Measure	Parents
Parental alliance	Parental Alliance Measure	Parents
Father- infant interaction	Naturalistic play	Father-infant
	CIB	
	P-PATS (subscale)	
Social support	SOS	Parents
Socioeconomic status	Questionnaire	Parents
Demographics	Questionnaire	Parents
Cognition	RIST	Parents
Social functioning	GAF	Mother
Newborn characteristics	APGAR	
	NNNS	
Transmission mechanisms		
Stress-exposure	Parenting Stress Index Short Form	Mother
	Perceived Stress Scale	Mother
	Hair samples/	Mother & infant
	Mass spectrometry analysis	
Stress-sensitivity	Saliva samples/	Mother
	Mass spectrometry analysis	
Caregiving representation	PCEQ	Parents
	CEQ	Parents
Mother- infant interaction	Naturalistic play	Mother-infant
	Still face	
	CIB	
	AMBIANCE	
	ICEP	
Obstetric complications	National registers/case notes	Mother
Infant outcome	Measure	
Infant neuromotor and cognition	NNNS	
	Bayley	
Infant behavior	CIB	
Infant attachment	Strange Situation Procedure	
Stress-sensitivity	Saliva samples/	
	Mass spectrometry analysis	
Infant health	National registers/case notes

**Table 2:** Schedule of assessments

Measure	Baseline	1–7 days	4 w	16 w	52 w
Mother					
Socio-demographics	X				
RIST	X				
SCID Lifetime mood and psychosis modules	X				
MADRS	X		X	X	X
BRMRS	X		X	X	X
PANSS	X		X	X	X
GAF	X	X	X	X	X
SAPAS	X				
AAP	X				
AAI	X				
SOS	X		X	X	X
Psychosis Attachment Measure	X				
ACES	X				
CEQ		X	X	X	X
PCEQ	X				
Cortisol Hair	X			X	X
Cortisol Saliva	X			X	X
Perceived Stress Scale	X			X	X
Parental Stress Index 3- Short Form				X	
Parental Alliance Measure				X	
Lifetimes diagnosis register/case notes					
Infant					
APGAR		X			
NNNS		X	X		
BSID_III-R				X	X
Cortisol Hair		X		X	X
Cortisol Saliva			X	X	X
Infant health from registers, case notes					X
Mother-infant interaction					
Naturalistic video interaction, CIB		X	X		
Still face/AMBIANCE/ICEP, CIB				X	
Strange Situation/AMBIANCE, CIB					X
Father					
RIST	X				
BSI-53	X			X	
AAP	X			X	
SOS	X				
PAM	X				
PCEQ	X				
CEQ		X	X	X	X
SAPAS	X				
Parental Alliance Measure				X	
Father-infant interaction					
Naturalistic video interaction, CIB, PPAT				X	

### Maternal risk

Lifetime and current psychiatric diagnosis will be confirmed using the psychosis and mood components of the Structured Clinical Interview for DSM-5 [16, 17].

Presence of personality disorder will be assessed using *The Structured Assessment of Personality, Abbreviated Scale* (SAPAS). It is an eight-item screening interview for personality disorders [18].

### Maternal predictors and moderators

#### Psychiatric symptom measures

We will also conduct a comprehensive assessment of level of psychotic, depressive and bipolar symptomatology across the clinical groups. These measurements will be taken routinely at baseline and follow-up points.

*The Montgomery Asberg Depression Rating Scale* (MADRS) [19] is a semi-structured interview designed to assess the presence and severity of 10 core symptoms of depression. It is a widely used measure of the severity of depressive symptomatology.

*The Bech-Rafaelsen Mania Rating Scale* (BRMRS) [20] is a structured interview scale for assessment of presence and severity of 11 core symptoms of hypomania/mania.

*The Positive and Negative Syndrome Scale* (PANSS) [21] will be used to measure psychotic and psychiatric symptoms incorporating positive, negative, disorganisation, excitement and emotional distress symptoms [22].

#### Social and occupational functioning

The *Global Assessment of Functioning* (GAF) [23] is a numeric scale (0 through 100) used by mental health clinicians and physicians to rate the social, occupational, and mental functioning of adults [24].

#### Cognitive screen

*Reynolds Intellectual Screening Test* (RIST) [25] is a short screening measure of general intelligence.

#### Social support

*The Significant Others Scale* (SOS) [26] determines the two main areas of operation of social support: “emotional support” and “practical support” for the six most important people in the respective social network, including their contact time with the infant.

*Parental Alliance Measure* is a questionnaire, which assesses the parenting aspects of the marital relationship. [27]

#### Maternal exposure to traumatic life events

*Adverse Childhood Experiences Study Questionnaires (ACES)* [28] is a 25-item self-report measure that assesses the breadth of exposure to childhood emotional, physical, or sexual abuse, and household dysfunction during childhood.

#### Demographics

We will collect routine clinical and socio-demographic information on age, ethnicity, education, deprivation, socio-economic status, occupation, number of people in the household (including other children), and receipt of medication.

#### Attachment

Maternal attachment classification is an important predictor of offspring attachment at 12 months [29–31]. Dismissing attachment patterns are overrepresented among adults with psychosis and bipolar disorder compared to other clinical and non-clinical groups [32–34], whereas preoccupied attachment are higher in depression than in non-clinical groups [34]. However, patterns of attachment in both mother and infant have never been investigated in a developmental high-risk sample incorporating non-affective psychosis, bipolar disorder and depression [35]. Thus we do not know whether the attachment relationship contributes to increased developmental risk amongst infants of mothers with SMI. However mothers with SMI and their infants may exhibit an insecure attachment relationship that significantly increases infant’s risk of psychopathology [36], whereas a secure mother-infant attachment relationship might protect against risk development in high risk infants. Therefore we seek to characterize attachment representations as a baseline predictor of early risk or resilient trajectories.

*The Adult Attachment Projective* picture system (AAP) [37] consists of eight drawings of attachment situations dealing with illness, solitude, separation, loss, and abuse, along with one neutral scene used as a “warm up”. The drawings are presented to the interviewee as a way of activating his/her attachment system. The narrative depiction of these drawings is transcribed and coded for attachment status by coders trained and reliable in the AAP coding system. The narratives are coded in terms of agency of self, connectedness in relationships, synchrony, defensive processing (avoidance or cognitive disconnection) and evidence of segregated systems. Subjects are also classified as secure, dismissing, preoccupied, or unresolved.

*The Adult Attachment Interview* (AAI, George, Kaplan & Main, 1987, unpublished manual) is a semi-structured interview, consisting of 20 questions and probes, allowing categorisation of an adult individual’s state of mind with regard to attachment. Each interview is transcribed verbatim and coded for attachment status by coders trained and reliable in the AAI coding system into four classifications: secure, dismissing, preoccupied, or unresolved (Unpublished manual, Version 7.1, Main, Goldwyn & Hesse, 2002).

*Psychosis Attachment Measure* [38] is a self-report measure assessing two dimensions of anxious and avoidant attachment. Total scores are calculated for each dimension by averaging item scores, with higher scores reflecting greater anxiety and avoidance.

### Paternal/significant other predictors and moderators

There is an increasing body of literature highlighting the impact both of paternal behavior on offspring development, and the contribution of the father’s mental state and availability to moderate the impact of maternal illness on the infant’s development and wellbeing [9, 39]. We have therefore included a short battery of measures for measuring paternal/significant other characteristics at baseline and follow-up (see Table 1 and 2 for measures applied). We have opted to leave the definition of significant other relatively broad to reflect contemporary social structures, including but not limited to fathers and kinship carers.

*Brief Symptom Inventory (BSI-53)* [40] is used to assess symptom level in the partner. It is a 53-item self-report inventory in which participants rate the extent to which they have been bothered in the past week by various symptoms.

Fathers and/ or significant others will also be invited to participate in a video recording while playing with the infant. The video recorded will last a period of 10 min.

*The Paternal-Physicality Affect and Touch Scale(PPAT),* subscale for excitatory arousal (Sethna, Murray and Ramchandani, 2008, Unpublished manual) is applied on video recordings of naturalistic father-infant play to assess paternal behavior.

### Infant predictors and moderators

Infant neonate characteristics will potentially impact the proposed transmission mechanisms with regard to postnatal maternal caregiving representation, mother-infant interaction and level of stress-exposure after birth.

*Neonatal Intensive Care Unit Network Neurobehavioral Scale* (NNNS) [41] is a 30-min, 128-item assessment of neurologic, behavioural, and stress/abstinence signs that evaluates the full range of infant neurobehavior [42].

### Transmission mechanisms

1. Stress exposure and transmission of stress-sensitivity.

Exposure to prenatal stress might lead to enduring changes in the infant’s neurophysiological regulation of stress [43] Early experiences such as relational stress can also influence the regulation of physiological responses to stress [44]. This can be measures by levels of cortisol in e.g. saliva. Previous findings indicate a transmission of *increased* cortisol levels from depressed mothers [45] and *lowered* levels from mothers with PTSD to their offspring [46]. Importantly deviations in maternal stress-sensitivity can contribute to stress in the mother-infant interaction. Low maternal cortisol has been proposed as a possible mechanism contributing to maternal difficulties in sensitively attuning to infant cues, which in turn impacts the infant’s reactivity towards, and recovery from stress [47]. Mothers whose interactions with their infants are most disrupted exhibit most deviation in cortisol levels [47].

*Cortisol Sampling.* Cortisol concentration in hair samples measure integrated stress exposure during the previous three months. Hair samples from mother and offspring will be collected three times during the study (see Table 1 and 2). Cortisol levels in hair samples from mother during pregnancy and from infant at birth indicate degree of infant exposure to prenatal stress. Saliva samples are collected three times across the study in relation to mild stressors in order to assess maternal stress-sensitivity. Each time three saliva samples are collected i.e. before the stressor (baseline), 20 min after (reaction) and 40 min after the stressor (recovery). Stressors are the AAI interview (baseline), the Still Face Procedure (16 weeks) and the SSP (52 weeks, see Table 1 and 2). Hair and saliva samples will be analysed using liquid chromatography–tandem mass spectrometry [48].

*The Perceived Stress Scale (PSS):* The Perceived Stress Scale [49, 50] is a 10-item self-report questionnaire focusing on perceived stress experience. Questions evaluate experiences of life being unpredictable, uncontrollable and distressing during the previous 30 days, and whether the respondent has been feeling nervous or stressed. Higher scores reflect higher degrees of perceived stress.

*Parenting Stress Index, 3rd Edition Short Form* (PSI/SF) is a 36-item self-report questionnaire [51]. It assesses level of stress related to the parental role and consists of three subscales: Parental Distress, Parent–child Dysfunctional Interaction and Difficult Child as well a scale for defensive responding. Parental stress is associated with negative parenting behaviors, e.g. lower levels of parental sensitivity and reciprocity, and high parental stress is seen among abusive and negligent parents [52].

2. Caregiving representation.

George and Solomon [53, 54] have proposed a mediating link between the mother’s attachment status and the attachment classification of her child, which is her symbolic representation of her relationship with her child i.e. her caregiving representation. Correspondence has been found between maternal caregiving representation, maternal adult attachment classification and the child’s attachment classification [55–57]. During pregnancy and the first 12 months of the child’s life, maternal caregiving representations are consolidated via maternal caregiving behaviors, in parallel to the development of the infant’s attachment behaviour. The caregiving representation reflects the mothers’ own attachment experiences, but her current life situation, actual level of social support and the relationship experience with the specific child is important in shaping her caregiving representation to each individual child. Caregiving *helplessness representations are* assigned to mothers who experience themselves as struggling but failing to manage or control both the child and their own negative emotions and is associated with infant disorganized attachment [58]. These findings suggest caregiving representation as an important mediator of intergenerational transmission of attachment and warrant further studies of the role of caregiving representation in transmission of risk and resilience from mothers with severe mental disorder to their infant.

*Caregiving Experiences Questionnaire* and *Prenatal Caregiving Experiences Questionnaire* (PCEQ & CEQ, Brennan, George, & Solomon, 2013, unpublished manual) is self-report measures assessing five forms of defensive processing that have been associated in the attachment literature with patterns of caregiving representation [53]. Three scales evaluate dimensions of organized caregiving representation—flexible integration, deactivation, and cognitive disconnection—as related to children’s secure, avoidant, and ambivalent-resistant/dependent attachment, respectively. The other two scales evaluate the dimension of caregiving dysregulation as related to disorganized infant attachment.

3. Mother–infant interaction.

Maternal sensitivity to infant cues during early interaction is considered important for infant development and has been associated with later attachment classification [59, 60]. From an intersubjective system theory approach both mother and infant contribute actively to shaping the interaction. From this approach, model of the dyadic regulation process taking place between an infant and a caregiver during interaction is referred to as the “Mutual Regulation Model” [61–64]. This model assumes that mother and infant through their interaction form a dyadic regulatory system, which regulates the infant’s biobehavioral organization, including regulation of negative emotions and stress. According to this model, normal development is a process of effective reciprocal social emotional communication, which is able to successfully repair episodes of mismatches i.e. temporary failures to regulated physiology, affect and stress. The successful repair of daily stressors leads to a cascade of positive affect, and homeostatic psychophysiology, whereas unsuccessful reparation lead to continuation of stress, cascades of negative affect and dysregulated psychophysiology e.g. increased physiological stress sensitivity [65].

Most studies of mother-infant interaction in severe mental disorder have been carried out in samples with depressed mothers. These findings indicate disturbed mother-infant interaction in depression [9, 66]. A few studies in bipolar disorder also indicate disturbed interaction in this population [67–69]. High risk schizophrenia studies show that early unstable family rearing conditions predict offspring diagnosis of schizophrenia [14, 70], but there has been a dearth of studies exploring early parent-infant interaction in schizophrenia, despite high-risk research [71] and clinical initiatives in this population [72]. Our own review work on early parent-infant interaction in schizophrenia [35] identified 27 studies from 10 cohorts, who comprised 208 women diagnosed with schizophrenia; 71 with other psychoses; 203 women with depression; 59 women with mania/bipolar disorder; 40 with personality disorder, 8 with unspecified mental disorder and 119 non-psychiatric controls. These cohorts comprised a mix of longitudinal and cross-sectional cohort studies and studies conducted within specialist Mother Baby Units. We identified consistent evidence of bias across the studies including selection, measurement, loss to follow-up, blinding of outcomes, confounding and statistical methods. Most studies included infants aged between 1- and 12 months. Data regarding neonates, ages 13–36 months and beyond 36 months were more limited. Those studies investigating the time period between 1- and 12-months found some evidence for maternal behavior in psychosis and schizophrenia, which differed from maternal behavior in the control sample. There was less evidence that infant behaviour differed from normal controls amongst offspring of maternal schizophrenia or maternal psychosis. Thus, previous research demonstrates disturbances in the mother-infant relationship and infant attachment in schizophrenia, bipolar disorder and depression indicating that interaction quality may affect infant development.

*Naturalistic video of mother infant interaction.* At the end of the NNNS at 1–7 days and 4 weeks, we will continue to video record for 5 min (*Naturalistic Video*) as the mother and infant reunite. This material will be used for coding CIB, see below.

*Still Face Procedure* by Tronick [73] will be applied at 16-weeks and will follow the specific procedures designed by Lyons-Ruth (personal communication) [47]. The testing session for mother–infant interaction is divided into three phases, Pre-Still Face, Still Face and Recovery phases, each of which had been explained to the mothers before the session began.

*Coding Interactive Behavior* (CIB) [44] is applied on video recordings of parent-infant interaction (reuion after NNNS at 1 and 4 weeks, Pre-Still Face and Recovery phase of the Still Face procedure, father-infant naturalistic play at 16 weeks, SSP at 52 weeks). It assesses parent, child and dyadic affective states and interactive styles. This measure is typically used with adults and children aged between 2 and 36 months, but can also be used for newborns. Subscales consist of six composites: parental sensitivity, intrusiveness and limit setting, child involvement, withdrawal and compliance, dyadic reciprocity, and dyadic negative state.

The ability of the mother-infant dyad to successfully repair episodes of mismatches will be measured by *The Infant and Caregiver Engagement Phases* (*ICEP*, Tronick, unpublished manual, personal communication). *ICEP* measures mismatching (rupture) and matching affective states (interactive repair) and are based on Tronick’ Monadic Phases Scoring System, Tronick and Weinberg’s Infant and Maternal Regulatory Scoring Systems (IRSS & MRSS), and Weinberg and Tronick’ work on affective configurations [74]. The ICEP-R phases combine information from the infant’s or caregiver’s face, direction of gaze and vocalizations. The ICEP-R engagement phases for the infant are Negative Engagement (further divided into withdrawn and protest), Object/Environment Engagement, Social Monitor and Social Positive Engagement. The ICEP-R codes for the caregiver are Negative Engagement (further divided into withdrawn, hostile and intrusive), Non-Infant Focused Engagement, Social Monitor/No Vocalizations or Neutral Vocalizations, Social Monitor/Positive Vocalizations and Social Positive Engagement.

The AMBIANCE measure is used to code disrupted caregiver behaviour during videotaped caregiver–infant interactions [75] (recovery phase of the Still Face procedure at 16 weeks, SSP at 52 weeks). The five dimensions of the AMBIANCE coding are affective communication errors, role/boundary confusion, fearful/disorientation, intrusive/negative, and withdrawing behaviour.

### Infant developmental outcome

#### Perinatal data, neuromotor and cognition

We will collect a range of data on neonatal outcome, informed by existing evidence of neonatal deviations in muscle tone and neuromotor weakness in high risk infant [76–78] using *Neonatal Intensive Care Unit Network Neurobehavioral Scale* (NNNS) [41]

Routinely available data including Apgar score, birth weight, gestational age, and obstetric complications will be collected from the mothers, national registers and case notes.

Given the established literature on developmental delays in cognitive and motor domains (e.g. [79–81]. we will assess development milestones using The *Bayley’s Scales for Infant Development 3rd Edition* (BSID-III) [82]. The BSID III items fall into the developmental areas of cognition, language and motor skills.

#### Stress-sensitivity

Increased stress-sensitivity has been proposed as a risk factor for development of psychosis and other severe mental disorder [83]. Stress-sensitivity might be inherited [83], a result of exposures during the prenatal period [43] or a result of postnatal stress. It has been associated to maternal stress and quality of mother infant attachment relationship [47, 83]. Infants with insecure and disorganized attachment classification have elevated cortisol levels during separation in the Strange Situation Procedure [84]. Infants classified as disorganized in attachment show greatest elevation and slowest return to baseline cortisol levels after SSP [85, 86]. They also differ from non-disorganized infants in diurnal cortisol rhythm, displaying a more flattened daily curve [45].

*Stress-sensitivity* is measured by saliva cortisol sampling in relation to mild stressors. Cortisol concentration is measured in saliva samples three times across the study. Each time three saliva samples are collected i.e. before the stressor (baseline), 20 min after (reaction) and 40 min after the stressor (recovery). Stressors are the NNNS examination (4 weeks), the Still Face Procedure (16 weeks) and the SSP (52 weeks, see Table 1 and 2).

#### Attachment

Attachment is an important domain of child socio-emotional development and is predictor for later psychopathology [87, 88].

*Strange Situation Procedure* (SSP) [59] will be used to assess the infants attachment and socio-emotional development. SSP provides a measure of the infant’s attachment behavior according to the four ways classification system: secure, avoidant, ambivalent/resistant and disorganized. In this procedure the infant is videotaped in a playroom during a series of eight structured 3-min episodes involving the infant, the mother, and a female stranger.

Our review [35] found evidence of greater attachment insecurity in offspring of mothers with psychosis compared to normal controls and greater avoidance compared to offspring of mothers with depression. Infant attachment data from offspring of mothers diagnosed with schizophrenia were assessed in three studies [89–91]. All used the Strange Situation Procedure (SSP) [84] for assessing infant attachment, however two studies used an abbreviated procedure (using only three or four of the eight episodes in the SSP). The third study, which used the full SSP found the largest proportion of insecure attachment in the schizophrenia group [89]. Only two ways (secure, insecure) and three ways (avoidant, ambivalent/resistant, secure) assessment of attachment type was carried through, whereas the additional disorganized attachment type, most clearly associated with psychopathology, was not assessed in psychosis. There are indications of and high rates of insecure and disorganized infant attachment in offspring of mothers with depression [66] and in offspring of mothers with bipolar disorder [67–69].

### Procedure

The study has been approved by The committees of Health research Ethics in the Captial Region of Denmark (Protocol no: H-2-014-024) and by the West of Scotland Research Ethics Service and the NHS GG&C Board Approval (REC Reference 14/WS/1051). The project is registered with ClinicalTrial.Gov (https://clinicaltrials.gov/ct2/show/NCT02306551). The study launched in October 2014 and the recruitment period is expected to be 3–4 years. Participants in Denmark are recruited from Region Zealand and Region Southern Denmark and in Scotland from Greater Glasgow and Clyde. Potential participants are identified by obstetric consultants screening referrals to obstetric wards and by midwifes at midwiferies. In addition perinatal mental health services and community mental health teams are invited to approach and refer participants to the study. Women in Scotland can also self-refer. All mothers with probable inclusion diagnoses will be approached for their consent to be referred to the research team. All those who consent to be referred to the research project will be approached for their informed and written consent by the research team. The diagnoses are confirmed by the research team, which carry out baseline data collection as well as follow-ups. This team consists of three PhD students, a postdoc and an assistant professor. If an inclusion diagnosis cannot be confirmed, the participant will be excluded from the study. The numbers of mothers with depression or without a psychiatric history are more numerous in services. In order to guard against introducing selection biases we will randomly select which mothers from these groups that are referred to the study. Randomization lists will be constructed electronically and will determine the order in which mothers, identified within a specific month, are approached for referral to and consent into the study. Once we have reached the monthly limit on participants from the mothers with depression or without psychiatric history recruitment from these groups will stop for that month.

### Sampling, power and proposed data analyses

Sample size is set to *N* = 200, with 50 participants in each group (inclusive of Denmark and Scotland). With this sample size, we can detect an effect size of 0.24 between groups (small to medium effect size) with a 5 % level of significance (alpha) and a power of 80 %.

Basic characteristics of the cohort will be addressed using descriptive statistics (means and standard deviations for numeric variables and frequencies for categorical variables). Informed consent rate will be addressed by estimating the inclusion rate along with a 95 % confidence interval using the standard central limit theory based approximation. In order to investigate infant risk outcome, associated risk factors and transmission mechanisms we will employ mixed effects modelling to account for the longitudinal structure of the data and repeated sampling. The mixed effect model can also without any extensions handle missing outcome data and hence the proposed analytical approach is robust towards loss to follow-up. If missing variables at baseline, against our expectation, surpasses 5 % we will employ multiple imputations before conducting the analyses described above. Directed Acyclic Graphs [92] will be used to identify pathways from baseline variables (e.g. diagnosis) to infant outcome measurements and important confounders.

## Discussion

The WARM study addresses the lack of knowledge about very early risk developmental status of infant of parents with severe mental disorder, specifically, the paucity of data on the impact of, and interaction between psychosocial risk and resilience factors for very early infant development. Patterns of attachment in both mother and infant, explored in the WARM study, have never been investigated in a developmental high-risk sample incorporating non-affective psychosis, bipolar disorder and depression.

Our study builds on developmental psychopathology concepts of multifinality [93] and focuses on three mechanisms for transmission of risk or resilience from parent to infant: stress-sensitivity, caregiving representation and quality of parent infant interaction as illustrated in Fig. 1. There is a need for long-term intervention studies, which explore the possibility of decreasing rate of mental illness in offspring [5]. We propose that this study will provide important data to inform early preventive strategies.

### Limitations

In this study, we aim to establish a cohort of pregnant women with SMI. This is a challenging group to recruit, and we will therefore recruit over a relatively long time period to optimize sample size. Our initial piloting indicates that important reasons for the difficulties in identification and recruitment are the demands of both severe mental disorder and pregnancy for inclusion and that the period for both identification and baseline assessment completion is limited to only a few months. This might lead to low recruitment rates and sampling bias. We estimate recruitment rates through systematic procedures for identification of potential participants, and we intend to explore sampling bias by registering recruitment rates, reasons for decline and by comparing our sample with register data of pregnant woman with SMI.
